# How aging of the global population is changing oncology

**DOI:** 10.3332/ecancer.2021.ed119

**Published:** 2021-12-13

**Authors:** Yan Fei Gu, Frank P Lin, Richard J Epstein

**Affiliations:** 1 New Hope Cancer Center, United Family Hospitals, 9 Jiangtai W Rd, Chaoyang, Beijing 100015, China​; 2 Garvan Institute of Medical Research, 384 Victoria St, Darlinghurst, Sydney 2010, Australia; 3 NH&MRC Clinical Trials Centre, 92 Parramatta Rd, Camperdown, Sydney 2050, Australia; 4 UNSW Clinical School, St Vincent’s Hospital, 390 Victoria St, Darlinghurst, Sydney 2010, Australia; a https://orcid.org/0000-0002-4640-0195

**Keywords:** clinical decision-making, demography, longevity, quality of life, supportive care

## Abstract

Population aging is causing a demographic redistribution with implications for the future of healthcare. How will this affect oncology? First, there will be an overall rise in cancer affecting older adults, even though age-specific cancer incidences continue to fall due to better prevention. Second, there will be a wider spectrum of health functionality in this expanding cohort of older adults, with differences between “physiologically older” and “physiologically younger” patients becoming more important for optimal treatment selection. Third, greater teamwork with supportive care, geriatric, mental health and rehabilitation experts will come to enrich oncologic decision-making by making it less formulaic than it is at present. Success in this transition to a more nuanced professional mindset will depend in part on the development of user-friendly computational tools that can integrate a complex mix of quantitative and qualitative inputs from evidence-based medicine, functional and cognitive assessments, and the personal priorities of older adults.

Epidemiologic shifts with impacts on health are occurring worldwide [1–3]. Life expectancies in most countries are rising, reflecting improvements in public health [4, 5], while birth rates are declining with urbanization [6–9]. This upending of the population age pyramid is now raising healthcare concerns due to an expected higher burden of age-related disabilities and diseases [10, 11] with consequent reduction in ‘health span’ [12, 13]. An inconvenient question is thus raised: does this global transition to a super-aging society [14–16] imply a need to change how medicine is practised today [17, 18]?

Cancer care is a timely focus for this debate [19]. Over the next forty years cancer will create the highest productivity burden of all disease groups as quantified by disability-adjusted life years, and will also overtake ischemic heart disease as the leading cause of death [20–22]. Even as age-standardized cancer mortality is declining due to better prevention, total cancer diagnoses and death rates are continuing to rise, with population aging the single main cause [23, 24].

Policies have been created to improve cancer care for older adults [25–27] in response to the realization that standard guidelines cannot define optimal management for all circumstances [28, 29]. Such guidelines have largely been based on the heuristic of late-phase randomized trial endpoints, especially overall survival [30], but there are weaknesses of this model from the viewpoint of older patients with cancer [31–33]. First, most trials have elected to treat younger and fitter patients with maximally tolerated drug doses, but by doing so have raised doubts whether the reported benefits are applicable to older people [34, 35]. Second, although randomized prospective trials are much respected in evidential terms, their credibility has been eroded by human factors. For example, despite the need to frame research questions with equipoise [36], positive results from clinical trials are favored by most involved parties – pharmaceutical companies [37], physicians [38], patients [39] and the press [40] – with only insurers and government agencies querying this tendency [41, 42].

Dominance of clinical research by industry [43] has likewise selected for biases due to non-publication of negative studies [44, 45], and trial designs favoring statistically significant outcomes [46, 47] – even though many such outcomes provide little real-world benefit to patients [48], especially if older [49]. This is consistent with the impression that average trial gains have tended to reduce in recent decades [50, 51], in part due to regulatory frameworks that have made therapeutic incrementalism the safest commercial strategy [52]. These caveats serve to remind us that clinical decisions should be made not by evidence alone, but by honest and self-critical discussion between doctors and patients [53], including older adults with cancer [54].

## How treatment decisions are becoming less influenced by chronological age

As the cohort of older adults with cancer enlarges, a widening spectrum of patient fitness *versus* frailty is to be expected [55], complicating therapeutic decision-making. A controversy often raised in this context is that the costliest phase of care tends to be the last year [56, 57], with even older patients often being prescribed anticancer therapy in the last month of life [58–60]. The solution to this would not seem to lie in implementing policies based on age, which is an unreliable predictor of health status [61], but in applying functional measures [62]. These include metrics of pre-morbid fitness – as approximated by estimated remaining life years without cancer [63] – and of daily activities or coping [64], as assessed by tools such as comprehensive geriatric assessment, which includes an evaluation of comorbidities [65–67].

**Figure 1.**
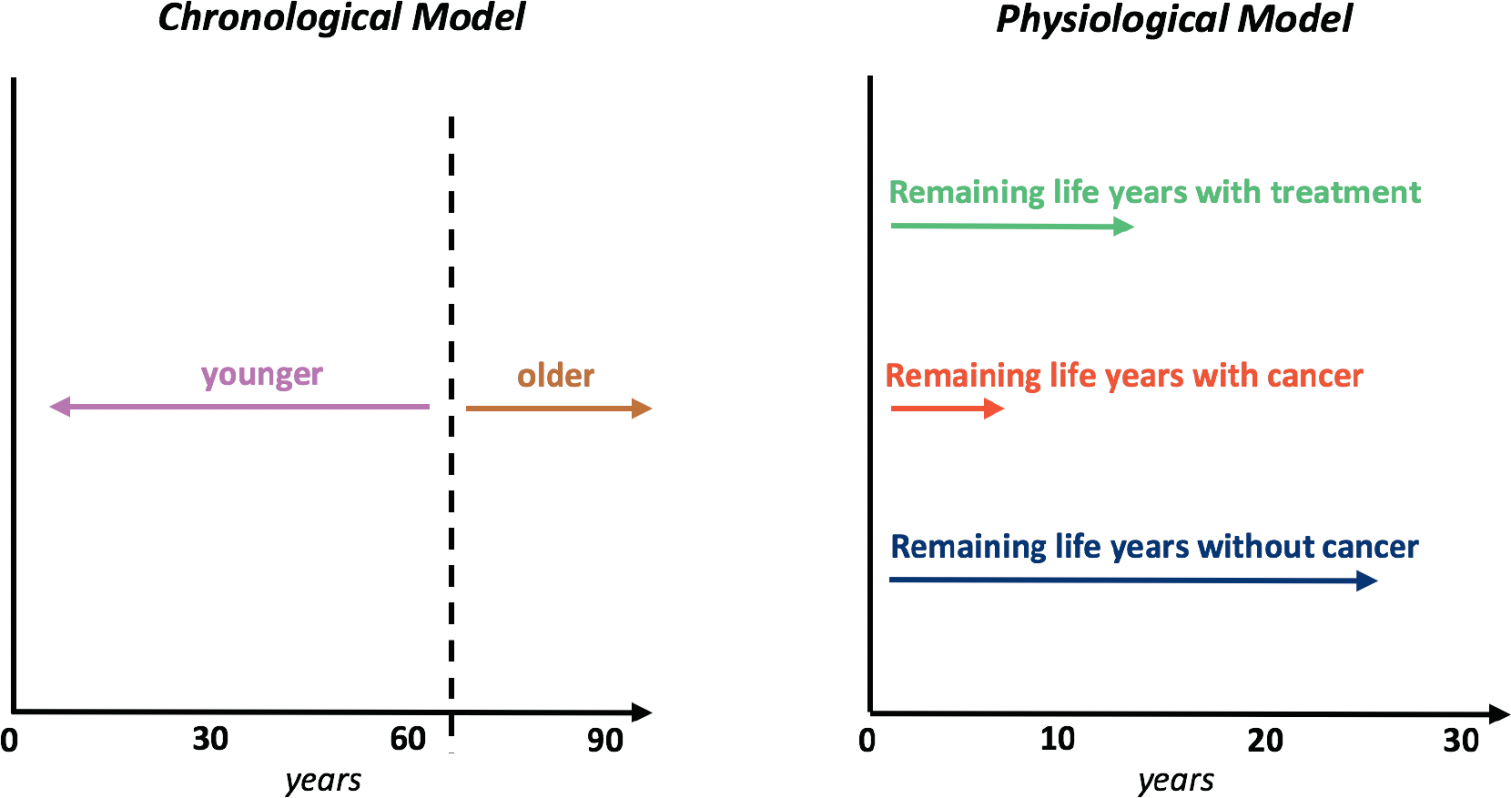
Comparison of the traditional chronological view of aging with a physiological (or “functional”, “biological”, etc.) viewpoint adapted for patients with a cancer diagnosis.

A “physiological age” so derived could be factored into evidence-based cancer and treatment expectations (e.g., estimated remaining life years with cancer, without and with treatment, plus quality of life with or without treatment] to create a decisional process that is not arbitrarily distorted by age [68, 69]. Assessing older patients with cancer in this function-based way should reduce risks of both over- and under-treatment [70, 71]. These risks include those from overreliance on the metric traditionally used by oncologists to assess fitness for drug trials, patient performance status [72]. In relinquishing these imperfect predictors of well-being, however, doctors will need to substitute more informative algorithms or endpoints (Figure 1).

## How cancer epidemiology is changing as populations are aging

Mortality from smoking-related malignancies is still rising, but these cancers should decrease as living standards and education improve [73]. In contrast, the epidemic of ‘lifestyle cancers’ due to overweight and under-exercise shows no hint of abating [74]. As population aging proceeds, lifestyle-related cancers may diverge into two strata: a younger group which more often has cardiovascular disease or diabetes, and an older group whose main risk factor is age alone [75]. The functional extremes, or stereotypes, of older adults with cancer – i.e., physiologically older or physiologically younger – illustrate the breadth of the wellness-illness spectrum in older individuals [76], highlighting the notion of health-related quality of life [77]. Physiologically older patients with cancer are by definition less fit than average for their age, based on non-cancer comorbidity [78, 79]. An estimate of physiological age is derivable by actuarial calculation of an individual’s likely death *x* years before or after the population’s mean life expectancy [80].

**Figure 2.**
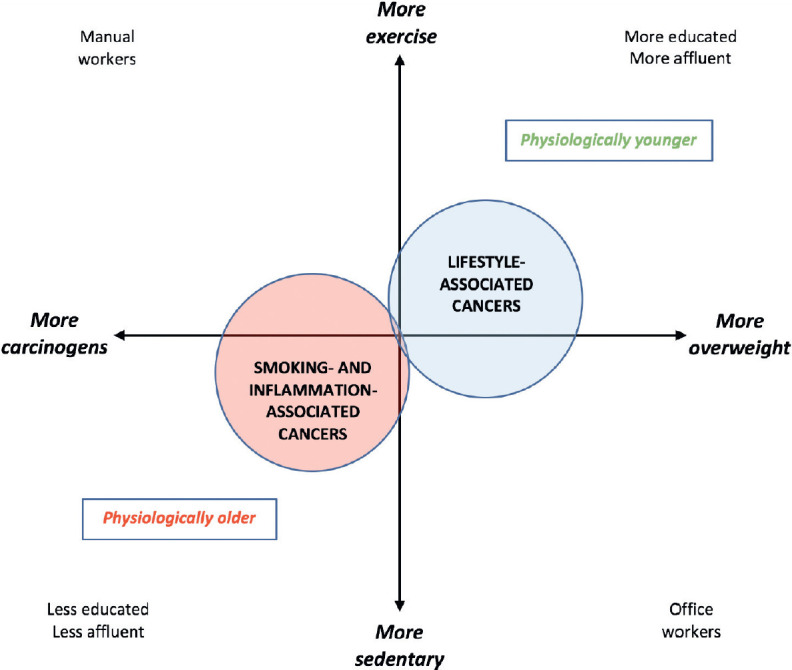
Pathogenetic and socioeconomic interplays relevant to the changing physiological age-specificity of cancer demographics.

Physiologically older *vs*. younger patients with cancer may also be separated by a wealth-health gradient that steepens with age [81]. The former cohort tends more often to be male, with more comorbidities, fewer financial resources, and more smoking- or inflammation-induced cancers, such as those of the lung, upper esophagus, stomach, bladder or rectum [82]. Physiologically younger patients tend to be more educated or affluent, and more prone to late-onset lifestyle malignancies such as those of the prostate, breast, endometrium, proximal colon, HPV-positive oropharynx, thyroid, or gastro-oesophageal junction; or melanoma, glioma or myeloma [74]. Since the latter patients tend to have fewer competing causes of death [83], they may in future come to comprise the dominant ‘older’ cohort (Figure 2).

## How fewer remaining life years are translating into more patient choices

Doctors have always modified treatment decisions on a holistic basis – for example, by deciding against morbid surgeries in favor of more conservative even if less curative interventions [84, 85], or by minimizing use of toxic chemotherapies [86]. Such decisions are often justified by patient frailty [31, 87]; however, non-frail older patients may also not receive the most effective therapies due to perceptions that benefits are less worthwhile in individuals with shorter life expectancy [88]. This is analogous to doubts over the use of cancer screening in older adults [89, 90], and represents one aspect of a controversy familiar to oncologists – namely, whether doing (or costing) more, in treatment terms, necessarily means doing better [91].

Demand will remain strong in all older patient groups for treatment modalities which are perceived, rightly or wrongly, to offer more hope of durable disease control with acceptable toxicity – e.g., molecularly-targeted drugs [92] and immune checkpoint inhibitors [93]. The spectrum of tumors in physiologically older patients tends to be more responsive to immunotherapies [94], even if only in a minority [95], whereas those in the physiologically younger group more often respond to hormonal or targeted drug therapies [96]. The higher educational profile of physiologically younger patients [97, 98] will likely favor interest in personalized oncology – i.e., targeted ‘smart drugs’ which are scientifically plausible [99] but not always empirically testable [100, 101]. So-called theranostic use of ^177^Lu-PSMA-617 radionuclide therapy to treat prostate cancer [102] is one example of a targeted treatment modality that may in future become more widely sought by some older patients – for example, when standard options are exhausted, or are anticipated to be poorly tolerated [103].

**Figure 3.**
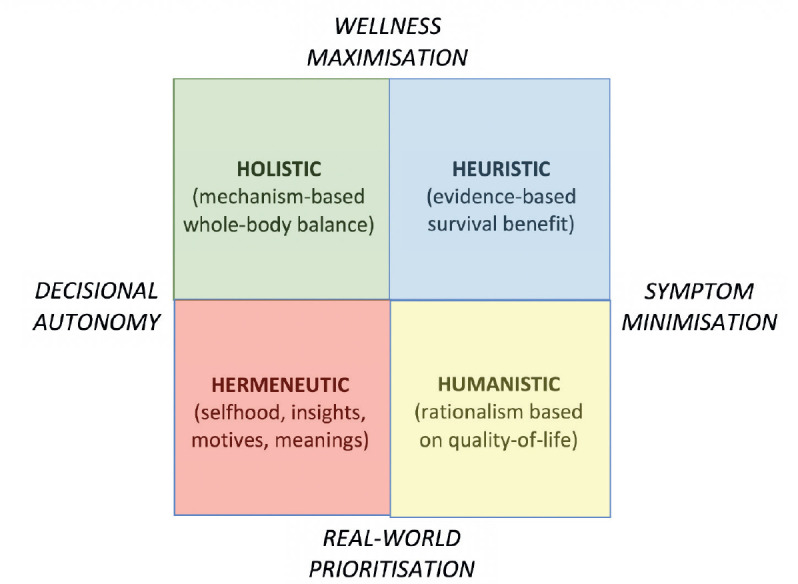
Decision-making schematic showing influence of survival gains balanced with other factors including mechanistic interventions, quality of life strategies, and personal priorities.

This ‘healthy aging’ cohort also seems likely to embrace mechanism-based non-pharmacologic health initiatives as anticancer interventions [104–106], with these including dietary modification – i.e., caloric restriction [107], fasting or weight loss [108], which inhibit insulin-like growth factor-1/Akt-mediated cancer cell survival [109] – and vigorous daily exercise, which blocks tumors via its effects on AMPK or mTOR signaling [110]. For young and fit patients similar to those recruited for trials, heuristic decision-making based on randomized trials showing survival benefit is appropriate, and for older patients such benefits also remain important [111]. Additional considerations may be prized by older adults, however [112]: these include humanistic prioritization of quality of life [113, 114], holistic balancing of mechanistic interventions with decisional autonomy [115–117], and hermeneutic notions of acceptance and finitude [118, 119] (Figure 3). Hence, one challenge is how to encourage older patients to include these value-adding endpoints in their decision-making without feeling that they are neglecting life-threatening survival priorities.

## How the needs of older patients with cancer are driving greater medical teamwork

The decision-making style of each older cancer patient will vary, ranging from independent to passive [120], but emotion remains a significant factor [121] with reassurance a frequent need [122]. This hints at the influence of incrementalist thinking among patients with cancer, such that even a one-month survival gain persuades many to request more toxic treatments [123], presumably due to fear [124]. Most patients worry about disease recurrence, and look to their oncologist as the ultimate defender against such contingencies [125, 126]; the mantra of whatever can be done will be done, and only the best is good enough, thus becomes a path of least resistance for physicians, and is further reinforced by litigation concerns. Such positivity appears to improve mood and quality of life for newly diagnosed patients with cancer by reducing prognostic awareness [127]. In the longer term, however, decision-making based on risk aversion and anticipated regret [128–130] – a response to threatened losses, as used by auctioneers to drive up offers from competing bidders [131, 132] – appears to undermine patient returns [133, 134].

This maladaptive positive feedback loop can be prevented by a team approach incorporating an initial discussion of goals [135]. By admitting fear factors and frequent sources of decision regret [136], older adults may become more able to consider a full range of coping options [137]. Early involvement of supportive, geriatric and/or mental health experts has thus become a standard of care [138-142] which is appreciated by older patients with cancer who value autonomy and dignity [143, 144]. For selected patients, religious professionals can also contribute to this team effort [145] – in part, perhaps, because religious people may accept better than doctors that death is a normal part of life, as distinct from its usual secular interpretation as a loss of life or an end to life [146]. Hence, as the age of cancer diagnosis rises, demand for professionals to work together is also likely to rise [147, 148], leading to more cross-disciplinary teamworking and better patient care.

To achieve this vision of a ‘geriatric tumor board’ [149], it will be ideal to develop computational algorithms able to integrate subspecialty metrics with individualised patient priorities as in Figure 3 [150]. By bridging these qualitative and quantitative dimensions, a validated algorithm could help to give older patients with cancer – plus their carers and doctors – confidence to move away from a cure-seeking to a more cost-effective cure-and-support-seeking culture [151]. As such, this could be a step away from the paradox that cancer costs rise further as knowledge grows, which trend is the opposite of that seen in technological progress as per Moore’s Law [152]. However, artificial intelligence approaches to even simple oncological decisions have still to mature [153], suggesting that a quantum leap in digital technologies will be needed to quantify subjective inputs [154]. Device-reported data [155, 156] for monitoring health outcomes in real time [157, 158] provide a fresh dimension of functional analysis for older adults with cancer, but this goal is still far from realization [159]. If and when innovative software eventually yields a systems medicine approach to shared decision-making [160, 161], utilization by third-party payers could create a virtuous pathway to better synergistic team care of older patients [162–165].

## Conclusions

The problems of the modern era have taught the importance of contingency planning. Population aging is a serious and imminent challenge for global healthcare cultures; treating an expanding group of older patients with widely varying fitness levels will demand deep changes to contemporary practices. Steps to ease this transition include the adoption of more informative patient metrics, greater use of function-based assessments, valuing and embracing the personal and spiritual priorities of older patients, and development of informatic infrastructures that can smoothly blend a mix of specialist assessments and patient preferences into 21st-century multidisciplinary cancer care.

## Conflicts of interest and funding

All authors declare no support from any organisation for the submitted work, no financial relationships with any organisations that might have an interest in the submitted work, and no other relationships or activities that could appear to have influenced the submitted work.

## Authors’ contributions

The manuscript was conceived and drafted by RJE, was read, criticised and revised through multiple drafts by YFG and FPL, and finally rewritten and approved by all authors. All authors are medical oncologists and PhDs, with FPL having additional expertise in medical informatics, and RJE in drug targeting and clinical trials. The clinical experience of the authors has taken place in Australia, China, Hong Kong, the UK, the USA and Singapore.
